# A study of chlorinated solvent contamination of the aquifers of an industrial area in central Italy: a possibility of bioremediation

**DOI:** 10.3389/fmicb.2015.00924

**Published:** 2015-09-02

**Authors:** Federica Matteucci, Claudia Ercole, Maddalena del Gallo

**Affiliations:** Laboratory of Environmental Microbiology, Department of Life, Health and Environmental Sciences, University of L’AquilaL’Aquila, Italy

**Keywords:** microbial bioremediation, anaerobic microcosms, organochlorine contamination, dense non-aqueous phase liquids (DNAPLs), geographic information system (GIS)

## Abstract

Perchloroethene, trichloroethene, and other chlorinated solvents are widespread groundwater pollutants. They form dense non-aqueous phase liquids that sink through permeable groundwater aquifers until non-permeable zone is reached. In Italy, there are many situations of serious contamination of groundwater that might compromise their use in industry, agriculture, private, as the critical case of a Central Italy valley located in the province of Teramo (“Val Vibrata”), characterized by a significant chlorinated solvents contamination. Data from the various monitoring campaigns that have taken place over time were collected, and new samplings were carried out, resulting in a complete database. The data matrix was processed with a multivariate statistic analysis (in particular principal component analysis, PCA) and was then imported into geographic information system (GIS), to obtain a model of the contamination. A microcosm anaerobic study was utilized to assess the potential for *in situ* natural or enhanced bioremediation. Most of the microcosms were positive for dechlorination, particularly those inoculated with a mineral medium. This indicate the presence of an active native dechlorinating population in the subsurface, probably inhibited by co-contaminants in the groundwater, or more likely by the absence or lack of nutritional factors. Among the tested electron donors (i.e., yeast extract, lactate, and butyrate) lactate and butyrate enhanced dechlorination of chlorinated compounds. PCA and GIS studies allowed delimiting the contamination; the microcosm study helped to identify the conditions to promote the bioremediation of the area.

## Introduction

Perchloroethene (PCE), trichloroethene (TCE), and other chlorinated solvents are widespread groundwater pollutants. They are among the most common pollutants at industrial sites. This is due to their extensive application in chemicals production, metal degreasing, and dry cleaning. These compounds are environmentally persistent and many pose serious health threats due to their toxic and sometimes carcinogenic effects (Henschler, 1994; Volpe et al., 2007). A major problem associated with the contamination of groundwater systems by these compounds stems from the formation of dense non-aqueous phase liquids (DNAPLs). PCE and TCE form DNAPLs that sink through permeable groundwater aquifers until a non-permeable zone is reached. The typical resulting distribution of the DNAPL is highly complex and non-uniform. Entrapped DNAPL mass tends to dissolves slowly into the flowing groundwater, serving as a long-term source of groundwater contamination (Aulenta et al., 2006). All these factors lead to an intense interest in the transformations of these compounds in the environment and in remediation processes (Ferguson and Pietari, 2000).

The geographic information systems (GISs) are currently among the most widely used tools used for managing and protecting the territory, as they are analytical tools with the ability to link different disciplines. These correlations between different topics are possible because the GIS software have the ability to georeference data, to link them through mutual spatial relations and to award descriptions of various kinds to individual data entered. The GISs are, therefore, georeferenced information management, analysis, and visualization systems, in which geographic information is handled through special format, called geographic dataset (Johnston et al., 2001). Spatial data of GIS consist of two components: (i) the geographical/geometric component, i.e., the spatial reference of the data, which can be a vector (points, lines, and polygons) or raster (set of pixels corresponding to the particular object); (ii) the statistical and textual component, with qualitative information describing the data.

Geostatistics is a set of statistical techniques used in the analysis of georeferenced data that can be applied to environmental contamination and remediation studies. Spatial statistical methods, of which geostatistics is a subset, are becoming increasingly popular, in part due to the availability of GIS software in a variety of application packages (Henshaw et al., 2004). In this study, the chlorinated solvents contamination at an industrial site in central Italy, is evaluated. Concern about the site and its future clean-up has triggered interest within the community because residential and commercial development surrounds the area.

The organo-halogenated pollution is now a problem for decades, but only in recent years we are starting to clarify its mechanisms through the study of their chemical and biological reactivity. Chlorinated solvents, however, once released into the environment, are subject to natural microbial degradation. They often behave as electron acceptors because of their substituents’ electronegativity, and are then reduced. The nature of halogen and the reaction conditions determine the redox behavior of organo-halogenated. In general, greater is the number of halogen substituents on a given molecule, higher is the oxidation state, and, therefore, greater is the ease with which it is reduced (Vogel, 1994). In particular, the reductive dehalogenation reaction is a process in which the halogen compound is reduced and a chlorine atom is replaced by one of hydrogen. It mainly occurs in compounds with a high number of halogen substituents, which are totally unaffected by aerobic microorganisms, such as PCE and TCE. In the natural course of their anaerobic biodegradation, they give rise to 1,2-*cis* dichloroethene (*cis* 1,2-DCE) and then to vinyl chloride (VC), a potent carcinogen, highly persistent, toxic and more mobile than others. They have maximum legal contamination levels in water ranging from 0.5 of VC to 1.5 μg/L of TCE and 1.1 μg/L of PCE (Decreto legislativo, 2006).

Many studies demonstrated that *Dehalococcoides* spp. is a bacterial genus known to completely dechlorinate the hazardous compounds PCE and TCE via DCE and VC to the terminal product, ethane (Aktaş et al., 2012; Löﬄer et al., 2013; Kranzioch et al., 2015). Recently, Cichocka et al. (2010) investigated on reductive dechlorination (RD) of tetrachloroethene to ethene. In this study, authors utilized a novel *Dehalococcoides*-containing culture that dechlorinates PCE to ethene from the contaminated anaerobic aquifer located in Bitterfeld (Germany). In this study, microcosms were prepared from groundwater and amended with lactate or hydrogen and PCE as an electron donor and acceptor, respectively. Poritz et al. (2013) have demonstrated the capability of two *Dehalococcoides mccartyi* strains to couple all reductive dehalogenation steps of PCE to ethene to growth. Poritz et al. (2013) demonstrated that the genome of *Dehalococcoides mccartyi* strain BTF08 encodes for 20 reductive dehalogenases, and is the first example of a genome containing all three enzymes that are necessary to couple the complete RD of PCE to ethene to growth.

During dechlorination, an increase in gene copy numbers of *pceA, tceA*, *and vcrA*, was observed (Poritz et al., 2013; Kranzioch et al., 2015).

The genes encoding TCE and VC reductive dehalogenases, are located within mobile genetic elements, suggesting their recent horizontal acquisition.

Microcosm studies provide information on natural attenuation processes in the contaminated aquifer, and on the capability of accelerating bioremediation processes, through the addition of amendments or microorganisms in the aquifer. Electron donors, such as lactic acid, butyric acid, and in particular, yeast extract (YE) are frequently added (Aulenta et al., 2005a; Cichocka et al., 2010). Indeed, in most of the cases, the hydrogen produced during fermentation of organic compounds is typically the direct electron donor for the RD (Di Stefano et al., 1991). A key point concerning the choice of fermentable organic substrates is the possible competition for hydrogen that can occur between dechlorinators and other H_2_-utilizing microorganisms such as methanogens (Fennell et al., 1997; Yang and McCarty, 2002). Previous studies demonstrated that dechlorinators have the potential to outcompete other H_2_-utilizers when H_2_ is present at low concentration, due to dechlorinator higher affinity for hydrogen (Smatlak et al., 1996; Ballapragada et al., 1997). Consequently, the utilization of substrates which are slowly fermented, such as butyrate or lactate, would result in a competitive advantage to dechlorinators over other H_2_-utilizers microorganisms (Fennell et al., 1997).

This paper describes a study of a chlorinated solvent contamination aquifer located in Central Italy.

All collected data were processed with a multivariate statistic analysis (in particular principal component analysis, PCA) and were then imported into GIS, to obtain a model of the contamination. Site characterization and groundwater-monitoring wells provided information to determine the extent and entity of contamination (Lee et al., 1998). An anaerobic microcosm study was utilized to assess the potential for *in situ* natural or enhanced bioremediation.

## Materials and Methods

### Field Site

**FIGURE 1:**
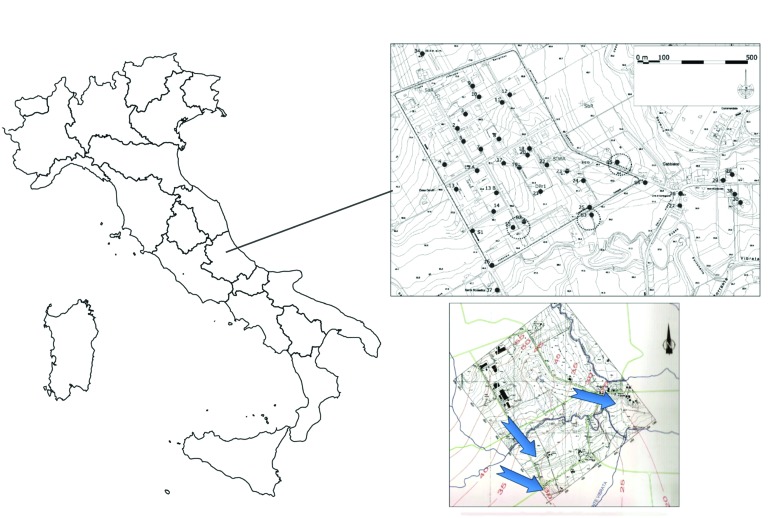
**Map of the industrial contaminated area showing wells (indicated with numbers) and coring locations S1, S2, S3, S4, and S5 (the ones used for the microcosms are circled).** Bottom right is the *Isopieze* map: the blue arrows indicate the direction of groundwater flow.

The sampling site is a large industrial area of a Central Italy valley located in the province of Teramo, morphologically developed between 40 and 75 m a.s.l., and located on a hilly area formed by the terraced alluvial deposits of the river Vibrata. The main industrial activities carried out in the area, present or past, are: the assembly of electronic circuits, sealing and testing of electronic circuits, soldering of electronic components, screen printing, packaging products, manufacturing chemical products for industrial use, textile fasteners, jeans sandblasting, production of leather bags and accessories, manufacture of iron and aluminum, storage truck, washing aggregates, production of burglar and fire alarm systems, leather wash, and wholesale trade in industrial machinery. The whole area is characterized by a significant contamination by chlorinated solvents, PCE and TCE in particular. The piezometric surface has been reconstructed recently. The “isopieze” map (**Figure 1**) shows that the direction of water flow was a trend toward Southeast.

### Aquifer Sample Collection

To obtain a representative sample of the aquifer under investigation, the manual sampling was carried out, using the manual method (disposable bailers). The various monitoring campaigns were focused on monitoring wells already present in the area, close to agricultural land, private residences, or industrial sites. To obtain significant analytical results we follow the recommendations of APAT (2006). Monitoring campaigns were carried out on 25 wells identified by numerical codes. The criterion by which we proceeded to the location of sampling points was based on a preliminary conceptual model, as suggested by EPA (2000). In particular, sampling sites were chosen in the direction of the water from upstream and downstream. Three locations, in three sites of the area and near the most contaminated wells, were chosen for soil and groundwater sampling, to set up microcosm bottles (**Figure 1**). Drillings, located according to the accessibility of places and pushed into the depth until reaching the impermeable layer below the aquifer, were performed with the method of drilling continuous core, with simple core barrel, dry and without drilling fluids. Soil samples derived from core drillings were taken from sections of saturated soil near the impermeable clay zone (where DNAPLs accumulate and, therefore, the greatest dechlorinating activity is generally present). Soil sections were then placed in a glass jars (2 L) and filled to the brim with groundwater from piezometers adjacent to the core drilling locations, to immediately ensure anaerobic conditions. Samples for headspace analysis was also prepared at this stage, taking 5 mL of water directly from the container through a disposable pipette, and introducing the water volume in a 10-mL vial, and then sealed. To avoid contamination by lab strains, all materials taken to the site from the lab were swabbed with ethanol before using.

### Gas-Chromatographic Analysis of the Aquifer Samples

Volatile organic halogenated compounds (VOX) determination in aqueous samples is usually carried out by gas-chromatography with electron capture detection (GC-ECD). Static headspace sampling (HS) is a well-known sampling technique for the analysis of VOX by GC (APAT IRSA-CNR, 2003). In HS, the sample is placed in a sealed vial, and heated in until the volatile compounds reach the equilibrium with the gas phase above the liquid. An aliquot sample of the gas phase is finally introduced into the column of the gas-chromatograph for final determination. HS for VOX has been adopted in several protocols by the United States Environmental Protection Agency (EPA). However, typical HS sensitivity achieved may not be enough for samples with very low analyte concentrations and static headspace stands only for the analysis of samples with relatively high-VOX concentrations, that is our case (Sanz-Medel et al., 2004). Static headspace was performed using an Agilent 6890N gas chromatograph. A “VOCOL^TM^” capillary column wall coated was used. Column length was 30 m, with an internal diameter of 0.53 mm and a stationary phase thickness of 3 μm. The detector utilized was an electron capture detector or ECD. Samples were prepared *in situ* at sampling moment to avoid any loss of analyte due to volatilization. The sample was prepared by introducing 5 mL of groundwater in a 10-mL vial fitted with a tightly sealed septum cap. Once in the laboratory, the vial was introduced in the headspace autosampler.

### Multivariate Statistical Analysis

The purpose of multivariate statistics is to make an exploratory analysis of the data matrix to quickly identify the correlations between variables and classify objects (samples or compounds) based on the characteristics of similarity/dissimilarity (Jolliffe, 2002).

Data collected from sampling campaigns were organized in the form of a matrix with *n* rows representing the objects (wells) and *p* columns representing variables (concentration of organo-halogenated compounds). The array matrix was properly converted into an ASCII file to apply multivariate analysis to our data. It was subsequently imported into the software PARVUS V-2008, which is a package of algorithms for multivariate statistical calculating. In this study, the application of V-NIPALS was utilized. It performs PCA on the imported dataset, using the algorithm non-linear iterative partial least squares (NIPALS). The result of PCA is represented by “scores”, “loadings”, and “biplot” graphs; they are two-dimensional graphs in which variables, objects, or both of them, are represented in the space of the first two principal components that together “explain” a significant proportion of the data variance.

### Making of GIS

As a result of monitoring campaigns, and related analytical determination of chlorinated organic compounds in groundwater, a complete database was obtained. It was characterized by informations on organochlorine compounds concentrations relating to each well considered at various stages of sampling. This database was then imported into GIS, creating a GIS capable of detecting a pattern of contamination.

Thematic isoconcentration maps were created, considering major chemical parameters that exceed the legal limits in groundwater of the area examined. The geographic coordinates of the wells were acquired with the GPS and then converted into plane coordinates reference system WGS84 – UTM, using the free software UltraSoft3D.

### Set-Up of Microcosms

Microcosms were prepared in 250 mL serum bottles with a soil suspension (70 mL) from the S2–S3 and S5 drillings of the contaminated site, by adding suitable electron donors and carbon source (lactate or butyrate 3 mM), YE 5 mg/L and a 86-mL of mineral medium (MM) containing: resazurin 0.1% w/v (redox indicator), NH_4_Cl, 0.5g/L; MgCl_2_⋅6H_2_O, 0.1 g/L; CaCl_2_⋅2H_2_O, 0.05 g/L; K_2_HPO_4_, 0.4 g/L; 5 mL of 5% w/v Na_2_S; 2 mL of 10% w/v NaHCO_3_; and a metal solution containing Nitrilotriacetic Acid (NTA, C_6_H_9_NO_6_), FeSO_4_⋅H_2_0, MnSO_4_⋅H_2_0, CoCl_2_⋅6H_2_O, ZnSO_4_⋅7H_2_O, H_3_BO_3_, NiCl2, and Na_2_MoO_4_⋅2H_2_O (Tandoi et al., 1994). The set-up of the experiment is described in **Table 1**. Each condition was in duplicate, to verify reproducibility. Some microcosms were inoculated with an anaerobic sludge (20 mL) from a wastewater treatment plant (Maymo-Gatell et al., 1995). Some microcosms were set up by adding contaminated groundwater instead of MM, to investigate the possibility of a natural attenuation in the area. Microcosms were sealed with Teflon-faced butyl rubber stoppers (Wheaton, Millville, NJ, USA) and aluminum crimp caps (Fennell et al., 2001). Bottles and caps were previously autoclaved to reduce emission of volatile organic compounds (Tandoi et al., 1994) and avoid microbial contamination. Headspace was flushed with a N_2_ (70% v/v) and CO_2_ (30% v/v) gas mixture, to ensure anaerobic conditions (Aulenta et al., 2007). Microcosms were incubated statically in the dark, at room temperature (18–22°C) for 1 year and monitored every 15 days. For the determination of chlorinated compounds and ethene, gaseous samples (100 μL) were removed from the headspace of microcosms using gas-tight syringes (Hamilton) and analyzed with a gas-chromatograph equipped with a capillary column and a mass spectrometer (GC-MS, Agilent series). It was assumed that DCE produced from more highly chlorinated ethenes was *cis*-DCE, which has a different calibration constant from *trans*-DCE due to a higher Henry’s constant (Gossett, 1987).

**Table 1:** Experimental scheme of microcosm bottles.

Microcosm samples	Electron donor	Anaerobic sludge	MM	YE	
				5 mg/L	200 mg/L
**Microcosm-S2 drilling**				
1	Abiotic control		•		
2	Biotic control		•		
3	Lactate 3 mM		•	•	
4	YE 3 mM		•		•
5	Butyrate 3 mM		•	•	
6	Lactate 3 mM	•	•	•	
7	Butyrate 3 mM	•	•	•	
**Microcosm-S3 drilling**				
8	Biotic control		•		
9	Lactate 3 mM		•	•	
10	Butyrate 3 mM		•	•	
11	YE 3 mM		•		•
12	Lactate 3 mM	•	•	•	
13	Butyrate 3 mM	•	•	•	
**Microcosm-S5 drilling**				
15	Biotic control				
16	YE 3 mM				•
17	YE 3 mM	•			•
18	Lactate 3 mM			•	
19	Butyrate 3 mM			•	
20	Butyrate 3 mM	•		•	

*S2, S3, and S5 drillings are circled in the map of schematic industrial contaminated area in **Figure 1**. MM indicates mineral medium. YE indicates yeast extract. YE was added as growth factor at the concentration of 5 mg/L and as electron donor at the concentration of 200 mg/L. Microcosm-S2 drilling (1–7) indicates microcosm bottles set-up with groundwater and soil from S2 drilling and MM. Microcosm-S3 drilling (8–13): indicates microcosms bottles set up with groundwater and soil from S3. Microcosm-S5 drilling (15–20): indicates microcosm bottles set-up with groundwater and soil from S5. Microcosms 1–13 were added with MM.*

On the sampled wells, the redox potential was measured. It ranged from -80 to 53.6 mV, which are good value for the potential reduction in the area. The redox potential of the groundwater samples used for the set-up of microcosms was: -11.2 mV for S2, 4.1 mV for S3, and 10.5 mV for S5. The redox potential was routinely checked inside the microcosms by checking the resulting color change of the resazurin.

## Results

The analytical results of chlorinated organic compounds were collected by different groundwater sampling campaigns and were combined with available historical data. All collected data showed that the area is characterized by a strong contamination, mainly due to compounds such as PCE, 1,1-DCE, and TCE, with concentration values that come to exceed even up to 1000 times over the legal limits.

### Gas-Chromatographic Analysis of the Aquifer Samples

**FIGURE 2:**
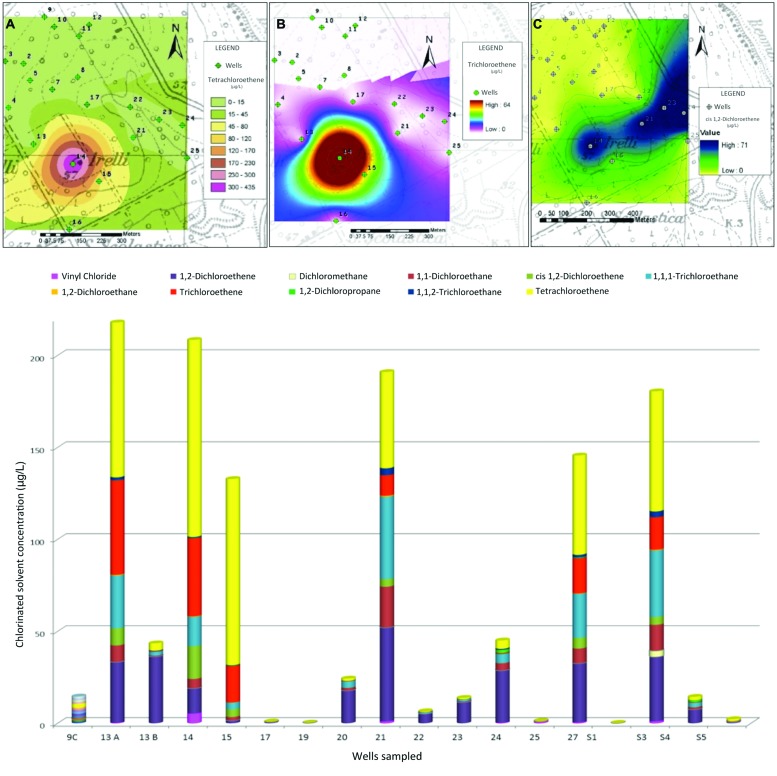
**Above: Isoconcentration maps of Perchloroethene (PCE) **(A)**, Dichloroethene (DCE) **(B)**, and 1,1-DCE **(C)**, Below: chlorinated solvents concentrations in sampled wells**.

Groundwater chemical analyses revealed the presence of a severe contamination by chlorinated solvents in the area. Compounds present at higher concentration were: PCE, TCE, 1,1,1-Trichloroethane (1,1,1-TCA), *cis*-DCE with traces of VC (**Figure 2**), and their concentrations far exceed the legal limits. Many of these compounds may be present in water as products of PCE degradation rather than by direct input, because they belong to its degradation chain. In addition, the compound most widely used in the site, especially in the past, at industrial level, appears, in fact, PCE.

### Principal Component Analysis

**FIGURE 3:**
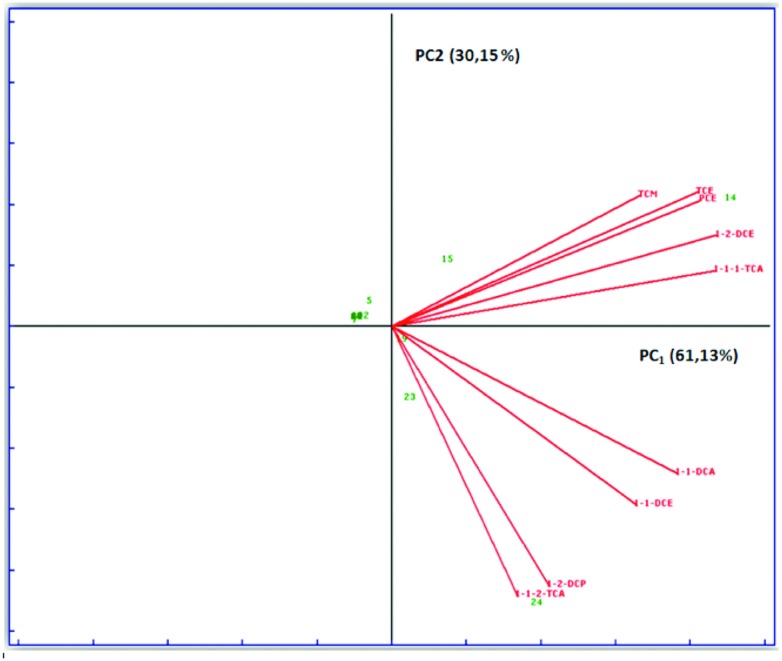
**Biplot graph (loadings ++ scores) simultaneously representing objects (wells) and variable (chlorinated solvents) in the space of two main components**. The *x*-axis shows the first principal component (PC1) that explains 61.13% of the original total variance data. The *y*-axis shows the second principal component that explains 30.15% of the original total variance data. The two principal components explain 91.28% of the original total variance data.

Several statistic tests were performed on the data matrix. The most significant are shown in **Figure 3**. PCA was performed on data relating to the different sampling campaigns considering as variables the halogenated solvent concentrations. Observing the **Figure 3**, it is clear that there is a strong correlation between PCE and TCE, which are linked to the same type of contamination, since the latter might also be originated from the degradation of the former. However, both come from similar sources or processes, both correlated to 1,2-dichloroethylene, belonging to the same anaerobic degradation pathway of PCE.

PCA was performed also considering as variables, in addition to the halogenated solvent, the other characteristic parameters of the aquifer: pH, conductivity, metals, etc. (results not shown). The target was to see if there were any correlations between contamination by chlorinated solvents and other types of contamination, or the intrinsic characteristics of the aquifer. The analysis showed that there was no significant correlation between organochlorine contamination and basic chemical characteristics of groundwater (such as pH, conductivity, or hardness), nor with the presence of metals and ions, because loadings of the organochlorine concentrations and those relating to other physical parameters are orthogonal each other.

### ArcGIS Software for Creating Isoconcentration Maps

Thematic isoconcentration maps were made using ArcGIS software on the values of PCE, TCE, and 1,1-DCE (main contaminants of the area), using the exponential model of interpolation “Kriging”, which gives a better match between the measured values and those predicted by the model. The aim was to have an overview of the contamination spread in the area and the achievement of potential receptors. The limits of such interpolation must be well brought out: although the sampled wells draw on the first aquifer under pressure, they have very different depth and flow, and unknown completion characteristics. The drawback, however, is sufficient to delineate the potentially contaminated area. **Figure 2** shows, respectively, the thematic maps of PCE, TCE, and 1,1-DCE. Organochlorine concentrations are shown in shades of colors; contamination originates from a specific point (coinciding with the probable source), and follows the course of the aquifer flow (**Figure 1**).

### Microcosm Study

The results of microcosm studies have provided information on natural attenuation processes in the contaminated aquifer, and on the capability of accelerating bioremediation processes, through the addition of amendments or microorganisms in the aquifer. These results are partial, because we need further data processing and a correlation with the molecular microbial composition of the microcosms. The electron donors were lactic acid, butyric acid, and YE. These substrates were compared both in the presence and in the absence of anaerobic MM and an anaerobic sludge. No microcosms prepared with groundwater only (microcosms from 15 to 20 in **Table 1**) showed any dechlorinating activity, even in the presence of lactate and butyrate (results not shown). In the abiotic controls and in the non-amended microcosms (microcosms 1, 2, and 8 in **Table 1**), dechlorination was not taking place and PCE was not degraded (results not shown). All microcosms prepared with YE only as an electron donor (microcosms 4 and 11 in **Table 1**), even in the presence of MM showed a low dechlorinating activity (results not shown).

**FIGURE 4:**
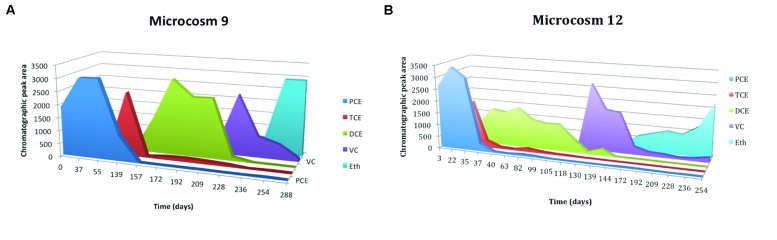
**(A)** Time course of reductive dechlorination (RD) of PCE in microcosms 9 amended with lactate and PCE, MM, and yeast extract (YE). **(B)** Time course of RD of PCE in microcosms 12 amended with lactate and digester sludge, PCE, MM, and YE.

In lactate-amended microcosms a complete degradation of PCE and TCE with formation of ethene occurs. **Figure 4A** shows the trend of microcosm 9, set up with soil and water from S3 drilling and amended with lactate. The dechlorinating activity began after a lag period of about 60 days, and led to a rapid accumulation of TCE, with the subsequent formation, by hydrogenolysis, of *cis*-DCE. The microcosm 3, amended with lactate, but set up with soil and water from S2 drilling, has the same trend (results not shown). **Figure 4B** shows the trend of microcosm 12, set up with soil and water from S3 drilling, with the addition of anaerobic sludge and amended with lactate. In this microcosm dechlorinating activity began after a lag period of about 20 days, and led to a rapid accumulation of TCE and subsequently of *cis*-DCE. DCE was gradually transformed into VC; ethene formation from VC was a slow process. However, at the end of the incubation all VC was converted to ethene.

**FIGURE 5:**
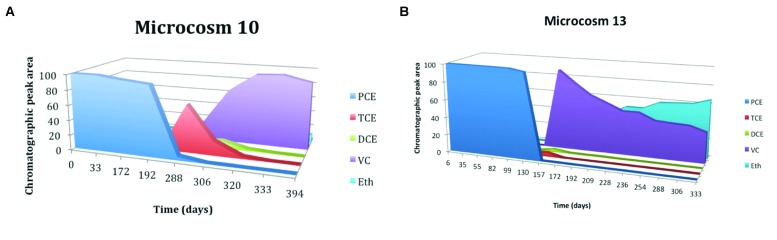
**(A)** Time course of RD of PCE in microcosms 10 amended with butyrate and PCE, MM, and YE. **(B)** Time course of RD of PCE in microcosms 13 amended with butyrate, and digester sludge, PCE, MM, and YE.

In butyrate-amended microcosms dechlorinating activity is lower. **Figure 5A** shows the trend of microcosm 10, set up with soil and water from S3 drilling and amended with butyrate. In this microcosm PCE began to be degraded after a lag period of about 4 months and there is a gradual accumulation of TCE, with the subsequent and fast formation of DCE. The concentration of TCE remained high for about 3 months, until both TCE that DCE were degraded to VC; with the decrease of VC concentration, slow formation of ethene occurred. The microcosm 5, amended with butyrate too, but set up with soil and water from S2 drilling, has a similar trend (results not shown). Microcosms bioaugmented with the anaerobic sludge dechlorinate PCE to *cis*-DCE, VC, and also ethene more quickly than the amended ones. **Figure 5B** shows the trend of microcosm 13, set up with soil and water from S3 drilling, with the addition of anaerobic sludge and amended with butyrate. Butyrate and digester sludge-amended microcosms had a shorter lag period than the respective treatments without digester sludge inoculum, but a greater lag period than lactate-amended with digester inoculum treatments. Microcosms 6 and 7 – added with digester sludge, amended with lactate and butyrate, respectively, and set up with soil and water from S2 drilling – had similar trends (results not shown).

## Discussions

The present study was carried out at a chlorinated-solvent-contaminated site in Central Italy. This site has a long history of groundwater pollution (over 20 years), most probably related to the various industrial activities occurred over the years. Groundwater analyses revealed the presence of high concentrations of PCE and also TCE, *cis*-DCE and VC (in traces), which are known to be PCE dechlorinating intermediates. The presence of *cis*-DCE suggests the occurrence of intrinsic dechlorinating activity at the site, because this compound is formed from the microbially catalyzed hydrogenolysis of TCE (Matturro et al., 2012). This hypothesis is confirmed by statistic and geostatistic analysis. From PCA, there is an obvious strong correlation between PCE, TCE and 1,2-DCE which are related to the same type of contamination: all compounds arise from the same type of industrial activity and belong to the same degradation chain. From PCA analysis it can be inferred also that contamination detected in groundwater is undoubtedly of exogenous origin, linked to the action of industrial activities in the area. GIS processing shows that the trends of isoconcentration curves and contamination plume of TCE and *cis*-DCE are very similar to those of PCA: depart from the same source and have a similar pattern along the groundwater flow. These are good basis for the applicability of a RD in the site. RD is a promising technology for bioremediation of PCE contaminated groundwater (Morse et al., 1997). *In situ* enhanced RD can be realized by stimulating the activity of dechlorinating microbial communities through the addition of electron donors to provide electrons required for PCE reduction (Aulenta et al., 2005b). We set up microcosm experiments to investigate the possibility of RD in the area. This study shows that the groundwater is not sufficient to start dechlorinating activity, while the addition of a MM with metal compounds and nutrients is necessary for the activation of the autochthonous microbial community (Lee et al., 1998). This experimental evidence underlines the importance of the metal complexes in the process of RD; reduction of halogenated aliphatic hydrocarbons take place concurrently to oxidation of some transition metals complexes such as cobalt, chromium, iron and nickel. These metals form the catalytic site of many biological enzymes. The negativity of abiotic controls indicates that no chemical and abiotic degradation of organochlorine is possible and the negativity of non-amended microcosms indicates that no natural attenuation is possible too. The microcosms prepared with YE only as an electron donor, showed a low-dechlorinating activity. This is in contrast with the literature in which the YE appears to be an effective electron donor (Di Stefano et al., 1991). Aulenta et al. (2005a) found that the addition of growth factors (i.e., YE and vitamin B_12_) caused a favorable on dechlorination. In our case, in fact, lactate and butyrate enhanced dechlorination with respect to the biotic control. Lactate-amended microcosms showed the shortest lag-phase (i.e., time prior to the onset of dechlorination) and the highest initial dechlorination rate; similar results were obtained by Aulenta et al. (2005b). As in other studies, highly chlorinated parent compounds are dechlorinated more quickly than their lesser chlorinated daughter products (Scheutz et al., 2014; Miura et al., 2015) The microcosms amended with lactate and butyrate showed a complete dechlorination, but the lactate seems to be the best electron donor. The adding of digester sludge, with a strictly anaerobic microbial community, led to a quicker dechlorinating activity compared to the amended treatments. As in other studies (Patil et al., 2014), the substantial dechlorination observed in these microcosms indicated that under the right nutrient conditions and microbial community dechlorination can proceed efficiently in environments also without classical dechlorinators bioaugmentation, such as *Dehalococcoides*. However, the developments of this works are the molecular study of the microcosms and of the presence of *Dehalococcoides* in indigenous microbial communities.

## Conclusions

The studies carried out in this research had different results.

The field work revealed a strong contamination of the area. Statistical analysis and geostatistics determined the possible presence of an initial degradation taking place in the site, thus the potential applicability of a natural or enhanced dechlorination.

From the results of the microcosm experiments, we deduced that natural attenuation could not be applied to the remediation of the area because of the absence of dechlorination observed in treatments reproducing the natural conditions present in groundwater (biotic controls and microcosms prepared with groundwater only). However, native dechlorinating populations are present in the soil of the contaminated site: all microcosms, amended with MM, and electron donors, showed dechlorinating activity. Dechlorinating activity of autochthonous microorganisms is favored by the presence of anaerobic MM and its absence represents a limiting condition for their metabolic dechlorinating activity. The addition of an anaerobic digester inoculum significantly accelerates degradation of chlorinated contaminants. Tested electron donors – lactate and butyrate, but not YE – stimulate the dechlorinating activity compared to the biotic control; the electron donor that stimulates the most dechlorinating activity is lactic acid, also in the presence of digester inoculum. This demonstrates that soil autochthonous microorganisms, if properly stimulated, may be able to perform all the stages of reductive dehalogenation of chlorinated ethenes up to ethene.

The present work needs to be completed with a molecular study, to investigate the microbial composition of the community and, specifically, the presence of dechlorinating bacteria known in the literature as capable of dechlorinating PCE to *cis*-DCE and to ethane such as *Dehalococcoides* sp. (DeWeerd et al., 1990; Krumholz, 1997; Holliger et al., 1998; Maymo-Gatell et al., 1999; Mészaros et al., 2013).

## Conflict of Interest Statement

The authors declare that the research was conducted in the absence of any commercial or financial relationships that could be construed as a potential conflict of interest.
